# Enhanced Fatty Acid Synthesis Leads to Subset Imbalance and IFN-γ Overproduction in T Helper 1 Cells

**DOI:** 10.3389/fimmu.2020.593103

**Published:** 2020-11-30

**Authors:** Shigeru Iwata, Mingzeng Zhang, He Hao, Gulzhan Trimova, Maiko Hajime, Yusuke Miyazaki, Naoaki Ohkubo, Yurie Satoh Kanda, Yasuyuki Todoroki, Hiroko Miyata, Masanobu Ueno, Atsushi Nagayasu, Shingo Nakayamada, Kei Sakata, Yoshiya Tanaka

**Affiliations:** ^1^The First Department of Internal Medicine, School of Medicine, University of Occupational & Environmental Health, Kitakyushu, Japan; ^2^Department of Hematology, The Fourth Hospital of Hebei Medical University, Shijiazhuang, China; ^3^Department of Immuno-oncology, The Fourth Hospital of Hebei Medical University, Shijiazhuang, China; ^4^Department of Clinical Subjects, High School of Medicine, Faculty of Medicine and Health Care, Al-Farabi Kazakh National University, Almaty, Kazakhstan; ^5^Research Unit/Immunology & Inflammation, Innovative Research Division, Mitsubishi Tanabe Pharma, Yokohama, Japan

**Keywords:** systemic lupus erythematosus, T-bet, IFN-γ, fatty acid synthesis, immunometabolism

## Abstract

Recent reports have shown the importance of IFN-γ and T-bet^+^ B cells in the pathology of SLE, suggesting the involvement of IFN-γ-producing T-bet^+^ CD4^+^ cells, i.e., Th1 cells. This study determined the changes in Th1 subsets with metabolic shift and their potential as therapeutic targets in SLE. Compared with healthy donors, patients with SLE had higher numbers of T-bet^hi^CXCR3^lo^ effector cells and T-bet^+^Foxp3^lo^ non-suppressive cells, which excessively produce IFN-γ, and lower number of non-IFN-γ-producing T-bet^+^Foxp3^hi^ activated-T_reg_ cells. These changes were considered to be involved in treatment resistance. The differentiation mechanism of Th1 subsets was investigated *in vitro* using memory CD4^+^ cells obtained from healthy donors and patients with SLE. In memory CD4^+^ cells of healthy donors, both rapamycin and 2-deoxy-D-glucose (2DG) suppressed T-bet^+^Foxp3^-^ cells, and induced T-bet^+^Foxp3^+(lo/hi)^ cells. Rapamycin induced IFN-γ-producing T-bet^+^Foxp3^lo^ cells accompanied with enhanced lipid metabolism, whereas 2DG induced IFN-γ-non-producing T-bet^+^Foxp3^hi^ cells. In memory CD4^+^ cells of SLE patients, inhibition of fatty acid synthesis, but not β-oxidation, suppressed IFN-γ production, and up-regulated of Foxp3 expression in T-bet^+^Foxp3^+^ cells. Metabolic regulators such as fatty acid synthesis inhibitors may improve the pathological status by correcting Th1 subset imbalance and overproduction of IFN-γ in SLE.

## Introduction

Systemic lupus erythematosus (SLE) is a common autoimmune disease. That nonspecific treatment of steroids or immunosuppressants is still used as the main therapeutic modality stresses the need to explore the pathological mechanisms and design new therapeutic strategies for SLE.

IFN signature plays an important role in SLE (1). In recent years, modular transcriptional repertoire analysis has demonstrated a different type I/II IFN signature and the importance of not only type I IFN but also type II IFN (IFN-γ) (2). While helper T (Th) cells are known to play an important role in the pathogenesis of SLE, their differentiation and functional abnormalities remain unclear (3).

In the 1990–2010, SLE pathology was discussed in the context of a balance between Th1 and Th2, together with the involvement of Th1 (4, 5). However, Th1 is only defined as an IFN-γ-producing cell, and other important markers for Th1 such as CXCR3 and T-bet, have not been simultaneously examined in these papers. Recently, the diversity of Th cells has been reported, and CXCR3, T-bet and IFN-γ have been reported to be expressed not only in Th1 but also in other diverse Th subsets. In particular, it was reported that T peripheral helper (TPH) cells and Th10 cells, which attract much attention and are important for B cell help, produced IFN-γ and expressed CXCR3 and T-bet (6, 7). Recent studies have emphasized the roles of T-bet^+^ B cells in SLE pathology, such as the production of autoantibodies and renal involvement (8–11). TPH cells and Th10 cells were known to be correlated with T-bet^+^ B cells in SLE (6, 12).

T-bet is known as a master transcription factor that regulates Th1 differentiation, and it was reported previously that T-bet plays a positive role in the regulation of IFN-γ production by effector Th1 cells (13, 14). Interestingly, various new functions of T-bet as a transcription factor have been recognized in recent years, including the presence of T-bet^+^Foxp3^+^ T_reg_, which specifically inhibits Th1 in mice (15) and inhibition of aberrant autocrine type I IFN and its downstream signaling in Th1 (16). However, the role of T-bet-expressing CD4^+^ cells, i.e., Th1 subsets, in SLE pathology remains unknown.

During the process of tissue infection, lymphocytes, including Th cells, rapidly change into the effector phase upon antigen stimulation. These activities require massive energy and rapid synthesis of biological components using amino acids, lipids, and nucleic acids (17). Recent studies in rodents have demonstrated enhanced glycolysis under aerobic conditions associated with such anabolic processes in the activation of various immunocytes (18–21). The differentiation regulation mechanism through immunometabolism involved in Th cell differentiation is being clarified in many studies mainly in mice; however, it is largely unknown in human, particularly in the pathology of autoimmune diseases, such as SLE.

The aim of this study was to shed light on the pathogenic process of SLE. Specifically, we determined the changes in Th1 subsets in SLE patients and the roles of these cells in the differentiation regulation mechanism *via* immunometabolism.

## Materials and Methods

### Cell Isolation and Differentiation

Peripheral blood mononuclear cells (PBMCs) were isolated from healthy adults using lymphocyte separation medium (Lympholyte-H; Cedarlane, Burlington, NC), and CD4^+^ T cells were purified by negative selection using CD4 T cell isolation kit (Miltenyi Biotec, Bergisch Gladbach, Germany) and magnetic separation (Miltenyi Biotec) for negative selection CD45RA^-^ memory T cells were further purified by negative selection using MACS Naive CD4^+^ T cell isolation kit; (Miltenyi Biotec). We confirmed that the purity of the obtained CD45RA^-^ memory CD4^+^ T cells was higher than 90%, as determined by flow cytometric analysis. CD45RA^-^ memory CD4^+^ T cells were activated by plate-bound anti-CD3 (2 μg/ml; eBioscience) and anti-CD28 (0.5 μg/ml; eBioscience) with anti-IFNGR Abs (10 μg/ml; R&D Systems, Inc.), tofacitinib (JAK inhibitor) (300 nM, kindly provided by Pfizer), rapamycin (mTORC1 inhibitor) (10 nM, Selleck Chemicals, Houston, TX), or 2-DG (3 mM, Wako Pure Chemical Industries, Osaka, Japan), C75 (racemic) (10 μM: Santa Cruz Biotechonology), Etomoxir (10 μM, 50 μM: Sigma Aldrich) cultured for 3 days in RPMI-1640 (Wako Pure Chemical Industries) supplemented with 10% FCS (Tissue Culture Biologicals, Tulare, CA), 100 U/ml penicillin and 100 U/ml streptomycin (Thermo Fisher Scientific, Carlsbad, CA).

### Patients

PBMCs were obtained from patients with SLE and healthy subjects. The clinical characteristics of the patients are summarized in **Table 1**.

**Table 1:** Baseline characteristics of the study subjects.

	SLE (n = 60)	HD (n = 31)	p-value
Age, years	41.4 ± 13.6	44.0 ± 19.3	0.8735
Gender	M:F 5:55	M:F 5:26	0.1366
Disease duration, months	142.3 ± 123.6		
Corticosteroid use	40/60		
SLEDAI score	11.5 ± 9.0		
BILAG score	12.9 ± 11.0		
BILAG A1 and/or B2	36/60		
clinical relevant organ involvement (BILAG A or B)			
constitutional	24/60 (40.0%)		
mucocutaneous	26/60 (43.3%)		
central nervous system	14/60 (23.3%)		
acute confusional state	4		
cerebrovascular disease	4		
seizure disorders	2		
headache	2		
mood disorder	2		
cognitive dysfunction	1		
aseptic meningitis	1		
neuropathy, cranial	1		
musculoskeletal	10/60 (16.7%)		
cardiovascular/respiratory	2/60 (3.3%)		
abdominal	4/60 (6.7%)		
renal	20/60 (33.3%)		
ophthalmic	0/60 (0%)		
haematological	20/60 (33.3%)		
anti-dsDNA Abs (IU/ml)	76.6 ± 124.9 (55.0%)		
anti-Sm Abs (U/ml)	40.4 ± 137.2 (10.9%)		
IgG (mg/dl)	1,798 ± 606		
CH50 (U/ml)	38.2 ± 17.2		
CRP (mg/dl)	0.8 ± 1.9		
ESR (mm/h)	47.2 ± 29.8		
WBC (/µl)	4,727 ± 2,040		
Lymph (/µl)	968 ± 668		
history of treatment			
Immunosuppressants	1.5 ± 1.6		
IVCY	20		
AZ	17		
TAC	19		
CsA	11		
MTX	8		
MZ	10		
RTX	1		
HCQ	7		
ABT	3		
MMF	1		
High-dose corticosteroid (times)	0.8 ± 0.8		
Steroid pulse (times)	0.5 ± 1.3		

Data are mean ± SD or number of patients.

See **Table 1** for abbreviations. IVCY, intravenous cyclophosphamide; AZ, azathioprine; TAC, tacrolimus; CsA, cyclosporin; MTX, methotrexate; MZR, mizoribine; RTX, rituximab; HCG, Hydroxychloroquine; ABT, abatacept; MMF, mycophenolate mofetil. High-dose corticosteroids were defined as the equivalent of prednisolone ≥0.8 mg/kg/day. Low-dose corticosteroids were defined as the equivalent of prednisolone ≤10 mg/day.

### Flow Cytometry

After washing the cells (PBMCs or CD4^+^CD45RA^-^ T cells), they were suspended in 100 ml of FACS solution (0.5% human albumin and 0.1% NaN_3_ in PBS) and stained with the following antibodies: V500-conjugated anti-CD4 Abs (#560769), PerCP-Cy™5.5-conjugated anti-CD25 Abs (#560503), FITC-conjugated anti-CD28 Abs (#555728), PerCP-Cy™5.5-conjugated anti-CXCR3 Abs (#580832), FITC-conjugated anti-CD38 Abs (#555459), and V500-conjugated anti-HLA-DR Abs (#561224) (all from BD PharMingen, San Diego, CA) for 30 min at 4°C. For intracellular staining of T-bet (V450)-conjugated labeled, #561312, BD PharMingen), Foxp3 (Alexa Fluor 488)-conjugated labeled, #560047, BD PharMingen), mTOR (pS2448) (#O21-404) (PE)-conjugated labeled, #563489, BD PharMingen), IFN-γ (APC)-conjugated labeled, #554702, BD PharMingen), PBMCs were fixed and permeabilized with Perm Buffer III (BD Phosflow™) or Transcription Factor Buffer Set (BD Biosciences) before intracellular staining, then analyzed on FACSVerse (BD Biosciences). Isotype-matched mouse IgG controls (BD, Phosflow) were used to evaluate the background. Finally, the cells were washed three times with FACS solution and analyzed with a FACSVerse (BD, San Jose, CA) and FlowJo software (Tomy Digital Biology, Tokyo). For IFN-γ staining, PBMCs were incubated with PMA (50 nG/ml, 1544-5, Wako), ionomycin (1 μg/ml, 10634, SIGMA-Aldrich, MO) and breferdin (2.5 μg/ml, B7651, SIGMA-Aldrich) for 1 h at 37°C.

### Lactate Assay

CD45RA^-^ memory CD4^+^ T cells were cultured alone or under stimulation/treatment for 3 days in 96-well plates. The culture medium was later collected and diluted properly for measurement of lactate concentration using Lactate Assay Kit II (BioVision, Milpitas, CA), and the protocol supplied by the manufacturer.

### Cytokine Production

IFN-γ, IL-2, IL-4, IL-6, and IL-17 levels in the culture media were determined by the BD Cytometric Bead Array human Flex set, according to the instructions provided by the manufacturer (BD PharMingen).

### Intracellular ROS Levels

After 3-day culture, CD45RA^-^ memory CD4^+^ T cells were gently washed twice with 37°C PBS, then incubated with 100 μl of 1X DCFH-DA medium solution at 37°C for 60 min. They were repeatedly washed thereafter with PBS at 37°C, then cultured for 5 min in wells each containing 100 μl of culture medium with 100 μl of 2X Cell Lysis Buffer. Finally, 150 μl of the culture mixture was transferred to a 96-well plate black culture fluorometric plates. Fluorescence was read with a fluorometric plate reader at 480/530 nm.

### Quantitative Real-Time PCR

Total RNA was prepared by using the RNeasy Mini Kit (Qiagen, Chatsworth, CA). First-strand cDNA was synthesized, and quantitative real-time PCR was performed in the Step One Plus instrument (Applied Biosystems, Foster City, CA) in triplicate wells of 96-well plates. TaqMan target mixes for *Enhancer of Zeste homolog 2 (*EZH2*)* (Hs00544830_m1), *basic leucine zipper transcription factor 2 (BACH2)* (Hs00222364_m1), *interferon regulatory factor 4 (IRF4)* (Hs01056533_m1), *PR domain containing 1 (PRDM1)* (Hs00153357_m1), carnitine palmitoyltransferase *IA* (*CPT1A*)(Hs00912671_m1), *carnitine palmitoyltransferase* *II* (*CPT2*)(Hs00988962_m1), fatty acid synthase (*FASN*) (Hs01005622_m1), sterol regulatory element-binding transcription factor 1 (*SREBF1*) (Hs01088679_g1), were purchased from Applied Biosystems. The mRNA expression level was normalized to the level of the endogenous control (*GAPDH* ribosomal RNA, #Hs99999905-m1, Applied Biosystems), and the relative quantity, compared with the PBMC sample as a reference, was calculated by using the quantification-comparative cycle threshold (DDCT) formula. The relative quantity was calculated by using the DDCT formula–referenced sample of PBMCs.

### Extracellular Flux Analysis

The XF96 Extracellular Flux analyzer (Seahorse Bioscience, North Billerica, MA) was used to quantify the oxygen consumption rate (OCR). CD45RA^-^ memory CD4^+^ T cells stimulated for 3 days were resuspended in XF media and then plated on XF96 cell culture microplates (2 × 10^5^ cells per well) coated with Cell-Tak (BD Biosciences). The OCR was measured in XF media (Agilent Technologies, Santa Clara, CA) supplemented with 1 mM sodium pyruvate, 10 mM glucose, and 2 mM l-glutamine under both basal conditions and in following the addition of 2 µM oligomycin, 2 µM carbonyl cyanide-*p*-trifluoromethoxy-phenylhydrazone (FCCP), and rotenone/antimycin A (Rot/AA).

### Electron Microscopy

Cells were collected and immersed in 2% glutaraldehyde solution at 4°C for 4 h. The samples were washed three times with 1 mol/L phosphate buffer, followed by 1% osmium tetroxide for 2 h, dehydrated in graded concentrations of alcohol (50, 70, 80, 90, and 100%), and embedded in epoxy resin. The embedded samples were then cut into ultra-thin serial sections (80 nm, Ultramicrotome, Leica UC-7, Leica, Germany) and stained with lead citrate and uranyl acetate. The samples were subsequently visualized using an electron microscope (JEOL, JEM-1200EX, Japan) at 80 kV at the Department of Electron Microscopy Center of University of Occupational & Environmental Health.

### Gas Chromatography Mass Spectrometry

2×10^5^ CD45RA^-^CD4^+^ cells metabolome under different conditions were extracted with 50% acetonitrile-water. Dried powder was dissolved in 20 mg/ml pyridine in methoxylamine-HCl, followed by N-Methyl-N-(trimethylsilyl)trifluoro-acetamide (MSTFA) reagent. The metabolome was identified using GC/MS (JMS-Q1500, 7890GC, JEOL, Japan) equipped with a direct capillary column DB–5MS sized 30 m×0.25 mm×0.25 μm film thickness. The column temperature was set at 80°C for 2 min and gradually increased at 15°C/min until it reached 320°C then held for 12 min. The temperature of the injector was adjusted to 230°C and the MS transfer line was adjusted to 250°C. Helium gas was used as the carrier gas, which was injected at a constant flow rate of 1.5 ml/min. In the next step, 1 μl of the sample was injected with a solvent delay at 2 min. An autosampler (7650A) was coupled with the GC and set in split mode for automatic injection of the samples and solvents. The effluent of GC column was transferred directly into the source of the MS through the transfer line. Electron ionization mass spectrometry fragments were initiated at 70 eV within the range of 30–500 m/z at a full scan mode. The temperature of the ion source was adjusted to 200°C. NIST mass spectral libraries were used to identify the obtained mass spectra of the active molecules in the extract.

### Statistical Analysis

All data were expressed as mean ± SD, unless otherwise indicated. Differences between groups were examined for statistical significance by the paired- or unpaired-*t* test. Pearson correlation coefficient was used to test the relation between two variables of interest. A *p*-value of <0.05 denoted the presence of statistical significance. Statistical analyses were conducted using the Prism software (Prism Software, Irvine, CA).

## Results

### Accumulation of IFN-γ Producing-CXCR3^lo^T-bet^hi^ Effector Memory CD4^+^ Cells in SLE

First, we used T-bet, CXCR3, and IFN-γ, which are originally known as typical markers of Th1 cells, to examine possible abnormalities of Th1 cells in the peripheral blood of SLE patients. **Table 1** summarizes the clinical background of the Japanese 60 participating patients. They included 5 males and 55 females, with a mean disease duration of 142 months, SLEDAI of 11.5 ± 9.0, BILAG 12.9 ± 11.0, clinical relevant organ involvement (lupus nephritis 33.3%, central nervous system 23.3%). We also enrolled 31 age- and gender-matched healthy donors (the control group) (**Table 1**).

**Figure 1:**
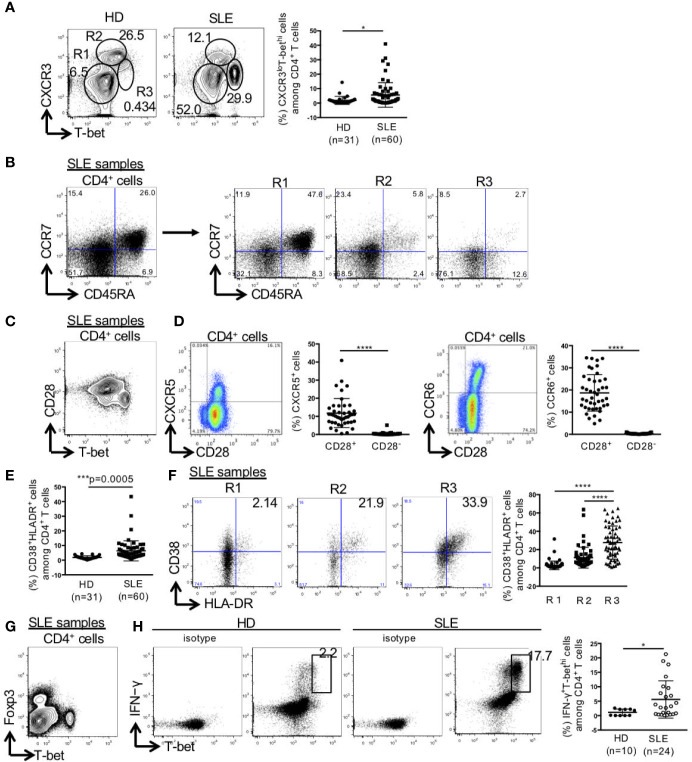
High percentage of CXCR3^lo^T-bet^hi^ effector memory CD4^+^ cells in patients with SLE. PBMCs were obtained from 31 healthy donors (HDs) and 60 SLE patients, and CD3^+^CD4^+^ T cells were gated. **(A)** Expression levels of T-bet and CXCR3 in CD4^+^ T cells were analyzed by intracellular staining using flow cytometry and shown in representative dot plots (left panel) and scatter plots of percentage of CXCR3^lo^T-bet^hi^ cells (right panel). **(B)** CCR7 and CD45RA were double-stained in CD4^+^ T cells and gated in R1 (CXCR3^-^T-bet^-^), R2 (CXCR3^hi^T-bet^lo^), and R3 (CXCR3^lo^T-bet^hi^) in CD4^+^ T cells in patients with SLE. **(C)** T-bet and CD28 in CD4^+^ T cells were double-stained in CD4^+^ T cells. **(D)** CD28 and CXCR5/CCR6 were double-stained in CD4^+^ T cells. Percentages of CXCR5^+^ cells in CD28^+/-^CD4^+^ cells and CCR6^+^ cells in CD28^+/-^CD4^+^ cells of SLE patients (n = 43) were shown in scatter plots. **(E)** Percentages of CD38^+^HLA-DR^+^ among CD4^+^ T cells were analyzed for HDs and SLE patients by flow cytometry and shown in the scatter plots. **(F)** Representative dot plots (left) and scatter plots (right) of expression of CD38 and HLA-DR in R1, R2 and R3 in CD4^+^ T cells from patients with SLE. **(G)** Expression of T-bet and Foxp3 in CD4^+^ T cells from patients with SLE was shown in the representative dot plots. **(H)** Representative dot plots of expression of T-bet and IFN-γ in CD4^+^ cells in HDs and SLE patients were shown (left). Percentages of T-bet^hi^IFN-γ^+^ cells among CD4^+^ T cells from HDs and SLE patients were shown in scatter plots (right). For T-bet and IFN-γ staining, PBMCs were incubated with PMA (50 ng/ml, ionomycin (1 μg/ml) and breferdin (2.5 μg/ml) for 1 h at 37°C. *p < 0.05, ***p < 0.001, ****p < 0.0001.

CD4^+^ T cells were separated into three populations of CXCR3^-^T-bet^-^, CXCR3^hi^T-bet^lo^, and CXCR3^lo^T-bet^hi^ cells. The percentage of CXCR3^lo^T-bet^hi^ cells among CD4^+^ T cells was significantly higher in SLE than the control (HD: 1.9 ± 2.8%, SLE: 5.6 ± 8.5%) (**Figure 1A**). CXCR3^-^T-bet^-^ cells mainly consisted of CCR7^+^CD45RA^+^ naïve cells, CXCR3^hi^T-bet^lo^ cells consisted of CCR7^+^CD45RA^-^ central memory cells and CCR7^-^CD45RA^-^ effector memory cells, and CXCR3^lo^T-bet^hi^ cells consisted mainly of CCR7^-^CD45RA^-^ effector memory cells (**Figure 1B**). CXCR3^lo^T-bet^hi^ cells which did not express CD28, were CXCR5^-^CCR6^-^ cells (**Figures 1C, D****)**. The percentage of HLA-DR^+^CD38^+^ cells among CD4^+^ T cells was significantly higher in SLE than the control. In addition, the percentage of HLA-DR^+^CD38^+^ cells among CD4^+^ T cells was significantly higher in CXCR3^lo^T-bet^hi^ cells than CXCR3^-^T-bet^-^ and CXCR3^hi^T-bet^lo^ cells in SLE (**Figures 1E, F****)**. CXCR3^lo^T-bet^hi^ cells were Foxp3^-^ cells (**Figure 1G**). CXCR3^lo^T-bet^hi^ cells showed high potential for IFN-γ production selectively in patients with SLE but not in the control (**Figure 1H**).

**Figure 2:**
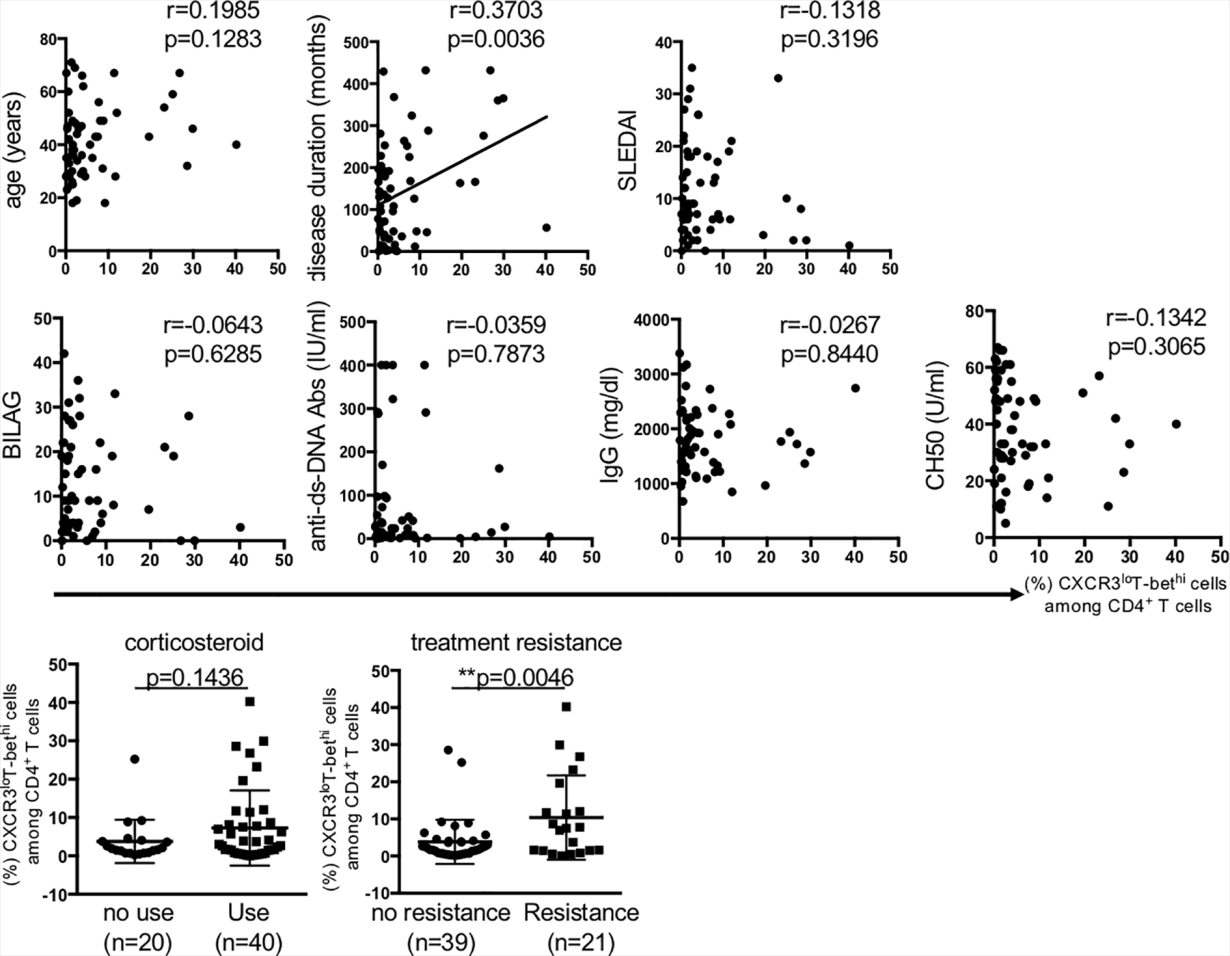
The relation of percentage of CXCR3^lo^T-bet^hi^CD4^+^ cells to clinical background in patients with SLE. Pearson correlation coefficient was used to test the relation between the percentage of CD4^+^CXCR3^lo^T-bet^hi^ cells and each factor. Treatment resistance was defined as lack of clinical response to ≥3 kinds of immunosuppressants and/or ≥2 re-increase in high-dose corticosteroid. SLEDAI; SLE disease activity score, BILAG; British Isles Lupus Assessment Group, ANA; Anti-nuclear antibody, Anti-Sm Abs; anti-Smith antibody, Anti-double stranded DNA antibody. The relation of the percentage of CXCR3^lo^T-bet^hi^ CD4^+^ cells to the factors of corticosteroid use and treatment resistance were analyzed using unpaired t-test. A *p*-value of <0.05 denoted the presence of statistical significance. Statistical analyses were conducted using the Prism software (Prism Software, Irvine, CA). **p < 0.01.

Next, we examined the relation of CXCR3^lo^T-bet^hi^ cells to the clinical background of patients with SLE. The results showed that disease duration and treatment resistance were the parameter that significantly related to the percentage of CXCR3^lo^T-bet^hi^ cells (**Figure 2**).

### High IFN-γ Producing T-bet^+^Foxp3^lo^ Non-Suppressive Cells and Low IFN-γ Non-Producing T-bet^+^Foxp3^hi^-Activated-T_reg_ Cells Percentages in SLE

**Figure 3:**
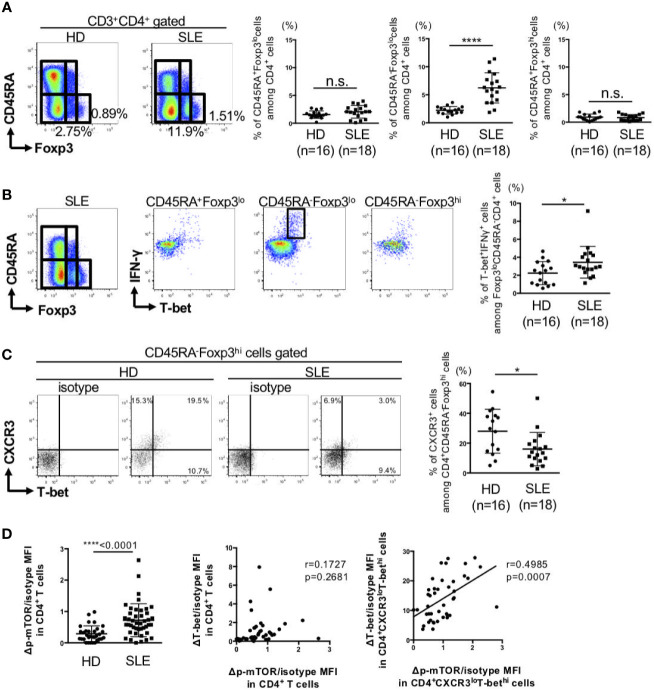
IFN-γ producing-T-bet^+^Foxp3^lo^ non-suppressive cells were increased, whereas IFN-γ nonproducing-T-bet^+^Foxp3^hi^ activated-T_reg_ cells were decreased in patients with SLE. PBMCs were obtained from 16 HDs and 18 SLE patients, and CD3^+^CD4^+^ T cells were gated. **(A)** Expression of Foxp3 and CD45RA in CD4^+^ T cells were analyzed by intracellular staining using flow cytometry and shown in the representative dot plots (left panels) and scatter plots of percentage of CD45RA^+^Foxp3^lo^ cells (naïve-Treg), CD45RA^-^Foxp3^lo^ cells (non-suppressive cells), and CD45RA^-^Foxp3^hi^ cells (activated-Treg) (right panels). **(B)** Double-staining of T-bet and IFN-γ expression in CD4^+^ T cells and gated in CD45RA^+^Foxp3^lo^ cells (naïve-Treg), CD45RA^-^Foxp3^lo^ cells (non-suppressive cells) and CD45RA^-^Foxp3^hi^ cells (activated-Treg) in HDs and SLE patients. Data are representative dot plots (left panels) and scatter plots of percentage of T-bet^+^IFNγ^+^ cells among Foxp3^lo^CD45RA^-^CD4^+^ cells (right panel). **(C)** Double-staining of T-bet and CXCR3 expression in CD4^+^ T cells and gated in CD45RA^+^Foxp3^hi^ cells (activated-Treg) in HDs and SLE patients. Data are representative dot plots (left panels) and scatter plots of percentage of CXCR3^+^ cells among CD4^+^CD45RA^-^Foxp3^hi^ cells (right panel). **(D)** The ΔMFI/isotype MFI of mTOR phosphorylation in CD4^+^ T cells from HDs and SLE patients was analyzed by flow cytometry and shown in the scatter plots. Right panel: Correlation between ΔT-bet/isotype MFI and Δp-mTOR/isotype MFI in CD4^+^ T cells and CD4^+^CXCR3^lo^T-bet^hi^ cells from SLE patients. Data are mean ± SD. Data are mean ± SD. *p < 0.05, ****p < 0.0001. n.s., not significant.

Next, we analyzed the changes in T-bet^+^Foxp3^+^ cells in SLE patients. The clinical characteristics of the patients are summarized in **Supplementary Table 1**. For this purpose, peripheral blood FoxP3^+^CD4^+^ cells were divided to three subsets of CD45RA^+^Foxp3^lo^ naïve-T_reg_, CD45RA^-^Foxp3^lo^ non-suppressive cells, and CD45RA^-^Foxp3^hi^ activated-T_reg_ (22). There were no differences in the percentages of CD45RA^+^FoxP3^lo^ naïve-T_reg_, CD45RA^-^FoxP3^hi^ activated-T_reg_ between SLE and the control. However, the percentage of CD45RA^-^FoxP3^lo^ non-suppressive cells was significantly higher in SLE than the control (**Figure 3A**). Considering in detail of these populations, the percentage of IFN-γ producing-T-bet^+^ cells among CD45RA^-^Foxp3^lo^ non-suppressive cells was higher while the percentage of IFN-γ non-producing-T-bet^+^ cells among CD45RA^-^FoxP3^hi^ activated-T_reg_ cells was lower in patients with SLE, compared to the control (**Figures 3B, C****)**.

Activation of mTOR, which is known to induce various anabolic processes, such as aerobic glycolysis, is important in the maintenance of a balance between T_eff_ and T_reg_ differentiation (23). Phosphorylation of mTOR in peripheral CD4^+^ T cells of patients with SLE was significantly higher than the control. Phosphorylation of mTOR was significantly correlated with T-bet expression in CXCR3^lo^T-bet^hi^CD4^+^ cells, but not in total CD4^+^ cells (**Figure 3D**). These results suggest that activation of mTOR can affect the imbalance between Th1 subsets.

### Rapamycin Induced IFN-γ-Producing-T-bet+Foxp3lo Cells, Whereas 2DG Induced IFN-γ-Non-Producing-T-bet+Foxp3hi Cells by Different Effects on Lipid Metabolism

**Figure 4:**
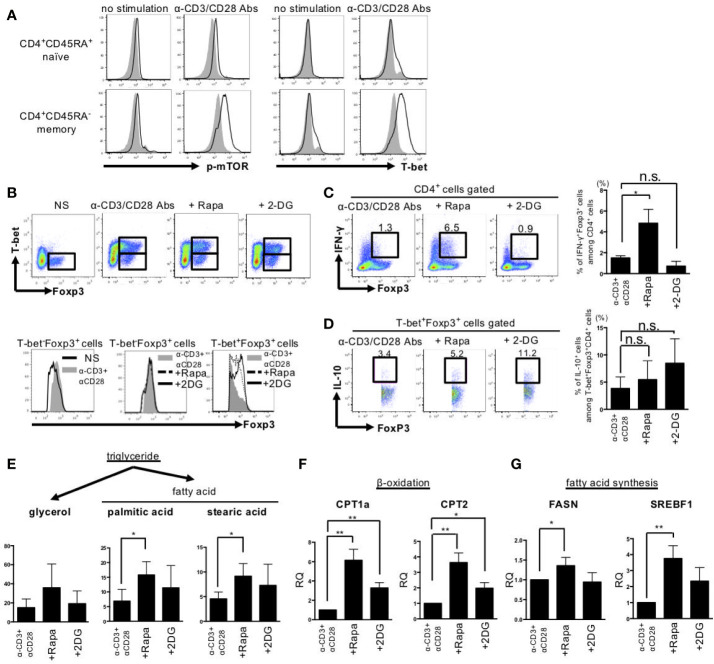
Rapamycin induced IFN-γ producing-T-bet^+^Foxp3^lo^ cells, whereas 2DG induced IFN-γ nonproducing-T-bet^+^Foxp3^hi^ cells by different effects on fatty acid metabolism. CD45RA^+^ naïve and CD45RA^-^ memory CD4^+^ T cells from peripheral blood of healthy donors were stimulated with anti-CD3 Abs and anti-CD28 Abs or rapamycin (10 nM) or 2DG (3 mM) for 72 h. **(A)** Representative results of mTOR phosphorylation and T-bet expression measured by intracellular staining and flow cytometry. **(B)** Upper panel; Expression of Foxp3 and T-bet in CD45RA^-^ memory CD4^+^ T cells after anti-CD3 Abs and anti-CD28 Abs stimulation with or without agents for 72 h. Lower panel; Foxp3 expression in gated regions (Left: T-bet^-^Foxp3^+^ cells in no stimulation or anti-CD3 Abs and anti-CD28 Abs stimulation, Middle: T-bet^-^Foxp3^+^ cells in anti-CD3 Abs and anti-CD28 Abs stimulation or with rapamycin or 2DG, Right: T-bet^+^Foxp3^+^ cells in anti-CD3 Abs and anti-CD28 Abs stimulation or with rapamycin or 2DG). **(C)** Data are representative results of Foxp3 and IFN-γ expression (further treated with PMA, ionomycin and Breferdin for 1 h) estimated by intracellular staining (left panel) and bar graph of the percentage of IFN-γ^+^Foxp3^+^ cells (right panel). **(D)** Representative dot plots of expression of IL-10 and Foxp3 in CD45RA^-^ T-bet^+^Foxp3^+^ cells treated with anti-CD3 Abs and anti-CD28 Abs and rapamycin or 2DG and bar graph of the percentage of IL-10^+^ cells among T-bet^+^Foxp3^+^CD4^+^ cells (right panel). Pooled data (mean ± SD of MFI) of three independent experiments in **(A–D)**. **(E)** The concentrations of glycerol, palmitic acid and stearic acid were measured by GC/MS. GC/MS data are mean ± SD of five independent experiments using cells from different healthy donors. **(F, G)** *FASN, CPT1A, CPT2* and *SREBF1* gene expression levels were determined by RT-PCR. These data are mean ± SD of four independent experiments using cells from different donors were shown. Data are mean ± SD. *p < 0.05. **p < 0.01. n.s., not significant.

In the next step, we examined *in vitro* the effects of rapamycin, a mTORC1 inhibitor, and 2DG, a glycolysis inhibitor, on cell metabolism, differentiation, and function in stimulated memory CD4^+^ cells obtained from HDs, as only a few CD4^+^CD45RA^-^ memory T cells were obtained from the patients with SLE. Stimulation with anti-CD3 Abs and anti-CD28 Abs induced mTOR phosphorylation and T-bet expression selectively in CD45RA^-^CD4^+^ memory cells (**Figure 4A**). The addition of rapamycin or 2DG reduced the percentage of CD4^+^CD25^+^Foxp3^-^ T_eff_ cells and increased CD4^+^CD25^+^Foxp3^hi^ T_reg_ cells among CD45RA^-^CD4^+^ cells, with a resultant significant increase in the T_reg_/T_eff_ ratio (**Supplemental Figure 1A**). *BACH2, IRF4, PRDM1, and EZH2* are transcriptional factors important for the differentiation and function of effector T_reg_ cells (24–27). Both rapamycin and 2DG induced upregulation of *BACH2, IRF4, PRDM1*, and *EZH2* in CD45RA^-^CD4^+^ cells (**Supplemental Figure 1B**). Evaluation of the changes in Th1, Th2, and Th17 cytokines demonstrated that rapamycin and 2DG had different effects on these cytokines, especially their effects on Th1 cytokines. For example, anti-CD3 Abs and anti-CD28 Abs-stimulated IFN-γ was more strongly inhibited by 2DG than by rapamycin. In contrast, interleukin (IL) 2 was not induced by anti-CD3 Abs and anti-CD28 Abs stimulation but strongly induced only by 2DG (**Supplemental Figure 1C**).

Next, we assessed the effects of rapamycin and 2DG on T-bet^+^Foxp3^+^ cells differentiation. Rapamycin induced IFN-γ-producing T-bet^+^Foxp3^lo^ cells. On the other hand, 2DG induced IFN-γ-non-producing T-bet^+^Foxp3^hi^ cells (**Figures 4B, C****)**. Both rapamycin-induced T-bet^+^FoxP3^lo^ cells and 2DG-induced T-bet^+^FoxP3^hi^ cells produced IL-10, and the production tended to be slightly larger in the latter (**Figure 4D**). Since rapamycin and 2DG have different effects on T-bet^+^Foxp3^+^ cell differentiation, we next examined the mechanism from the perspective of cell metabolism using CD4^+^CD45RA^-^ memory T cells obtained from HDs. Although rapamycin and 2DG had similar effects on the dynamics of cellular metabolism, such as aerobic glycolysis, mitochondrial function and glutaminolysis (**Supplemental Figures 2****,** **3**), their effect on lipid metabolism was different. The expression levels of fatty acid oxidation-related enzymes such as *CPT1a* and *CPT2* were increased by both rapamycin and 2DG, while the expression levels of fatty acid synthesis-related enzymes such as *FASN* and *SREBF1* were more highly increased by rapamycin compared with 2DG. This effect of rapamycin was coupled with larger increases in palmitic and stearic acid levels compared with 2DG, suggestive of enhancement of lipid metabolism, selectively in fatty acid synthesis (**Figures 4E–G**). These results indicate that rapamycin, but not 2DG, enhanced lipid metabolism, resulting in distinct effect in T-bet^+^Foxp3^+^ cell differentiation and its IFN-γ production.

### Inhibition of Fatty Acid Synthesis in Memory CD4+ Cells Obtained From SLE Patients Resulted in Suppression of IFN-γ Production and Up-Regulated Foxp3 Expression in T-bet+Foxp3+ Cells

**Figure 5:**
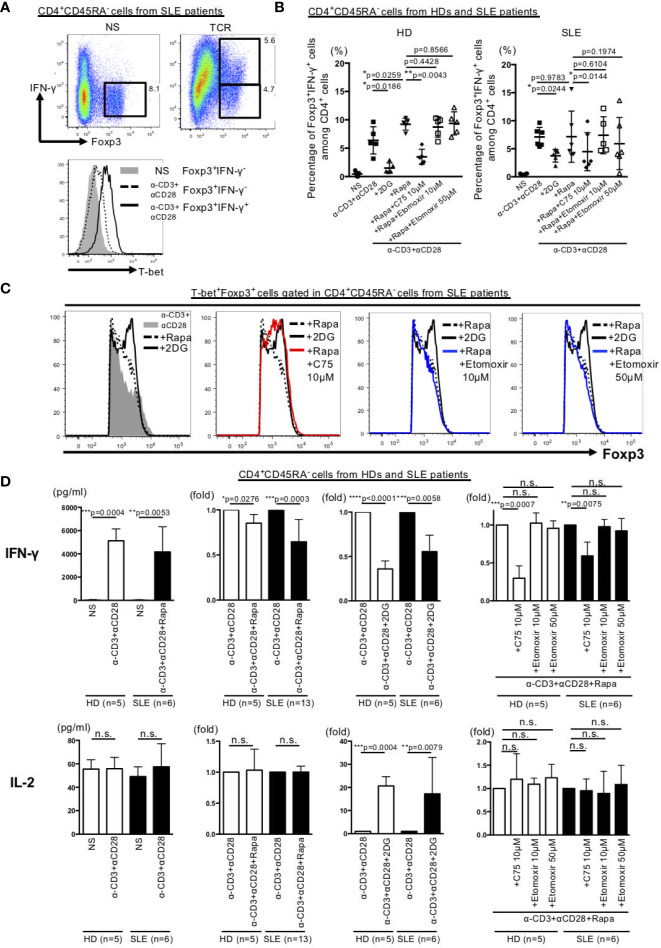
Inhibition of fatty acid synthesis suppressed IFN-γ production and increased Foxp3 expression in T-bet^+^Foxp3^+^ cells in memory CD4^+^ T cells in SLE. CD45RA^-^ memory CD4^+^ T cells from peripheral blood of HDs and patients with SLE were stimulated for 72 h with anti-CD3 Abs and anti-CD28 Abs or with 2DG (3 mM), rapamycin (10 nM), C75 (10 μM) or etmoxir (10 or 50 μM). **(A)** Expression of Foxp3 and IFN-γ in CD45RA^-^ memory CD4^+^ T cells from patients with SLE. Representative experiment depicting Foxp3 and IFN-γ expression in SLE samples (upper panel) and histogram of T-bet expression (lower panel) in three independent experiments. **(B)** Bar graph of the percentage of Foxp3^+^IFN-γ^+^ cells among CD45RA^-^CD4^+^ cells from HDs and patients with SLE. Data are mean ± SD of three experiments using CD45RA^-^ memory CD4^+^cells from each six different donors of HDs and SLE patients (paired T test). **(C)** Representative data of Foxp3 expression gated in T-bet^+^Foxp3^+^ cells in CD4^+^CD45RA^-^ cells from SLE samples in three independent experiments. **(D)** Concentration and fold change of cytokines by cytometric bead array in HDs and SLE samples. Data are mean ± SD of using CD45RA^-^ memory CD4^+^cells from each different donors of HDs and SLE patients (paired T test). *p < 0.05. **p < 0.01. ***p < 0.001. ****p < 0.0001. n.s., not significant.

We also investigated the role of dyslipidemia in the observed changes in T-bet^+^Foxp3^+^ cell differentiation and IFN-γ production by memory CD4^+^ T cells of HDs and SLE patients. The results showed that stimulation of the memory CD4^+^ T cells with anti-CD3 Abs and anti-CD28 Abs induced IFN-γ-producing T-bet^+^Foxp3^+^ cells in SLE samples (**Figure 5A****)**. Furthermore, the addition of rapamycin increased IFN-γ-producing T-bet^+^Foxp3^+^ cells in HDs cells. However, unlike the results in HDs cells, IFN-γ-producing T-bet^+^Foxp3^+^ cells remained unchanged by the addition of rapamycin in SLE patients (**Figures 5B, C**). Moreover, IFN-γ-producing T-bet^+^Foxp3^+^ cells did not change following the addition to rapamycin of etomoxir, a fatty acid β oxidation inhibitor, but was significantly inhibited by C75, a fatty acid synthesis inhibitor in both HDs and SLE patients (**Figure 5B**, **Supplementary Figure 4A**). The addition of C75 to rapamycin increased Foxp3 expression in T-bet^+^Foxp3^+^ cells to a level similar to that seen with 2DG (**Figure 5C**, **Supplementary Figure 4B****)**.

Finally, we evaluated the effects of various metabolic regulators on the production of IFN-γ and IL-2, which are two Th1 cytokines produced by memory CD4+ T cells in HDs and SLE. IFN-γ production induced by anti-CD3 Abs and anti-CD28 Abs stimulation was inhibited by rapamycin and 2DG and further inhibited by the combination of C75 plus rapamycin in both HDs and SLE patients (**Figure 5D**, **Supplementary Figure 4C****)**. Interestingly, 2DG, but not anti-CD3 Abs and anti-CD28 Abs stimulation, induced IL-2 production in both HDs and SLE patients (**Figure 5D****)**.

## Discussion

We investigated the changes in Th1 subsets in SLE and their involvement in SLE pathology. Our results showed abundance of T-bet^hi^CXCR3^lo^ effector cells and T-bet^+^Foxp3^lo^ non-suppressive cells (which produce large amounts of IFN-γ) in SLE, compared with deficiency of T-bet^+^Foxp3^hi^ activated-T_reg_ cells (which do not produce IFN-γ). These changes were considered to be involved in treatment resistance. In the *in vitro* arm of the study, we showed that treatment of stimulated memory CD4^+^ cells with rapamycin and 2DG resulted in suppression of T-bet^+^Foxp3^-^ cells and induction of T-bet^+^Foxp3^+(lo/hi)^ cells. Interestingly, rapamycin alone enhanced lipid metabolism and induced IFN-γ-producing T-bet^+^Foxp3^lo^ cells, whereas 2DG induced IFN-γ-non-producing T-bet^+^Foxp3^hi^ cells. In memory CD4^+^ cells of SLE patients, inhibition of fatty acid synthesis suppressed IFN-γ production and enhanced Foxp3 expression in T-bet^+^Foxp3^+^ cells. Thus, our results demonstrated that SLE is associated with IFN-γ overproduction by Th1 cells and Th1 subset imbalance due to metabolic abnormalities including enhanced fatty acid synthesis.

In the 1990–2010, SLE pathology was discussed in the context of a balance between Th1 and Th2, together with the involvement of Th1 (4, 5). However, Th1 is only defined as an IFN-γ-producing cell, and other important markers for Th1 such as CXCR3 and T-bet, have not been simultaneously examined in these papers. Recently, the diversity of Th cells has been reported, and CXCR3, T-bet, and IFN-γ have been reported to be expressed not only in Th1 but also in other diverse Th subsets. In particular, it was reported that TPH and Th10, which attract much attention and are important for B cell help, produced IFN-γ and expressed CXCR3 and T-bet (6, 7). In our study, the simultaneous confirmation of CXCR3, T-bet, and IFN-γ expression in CD4^+^ cells revealed that CXCR3^lo^T-bet^hi^ cells, rather than CXCR3^hi^T-bet^lo^ cells, overproduced IFN-γ and was closely involved in the pathogenesis of SLE, including treatment resistance (**Figures 1**, **2**). In addition, our results suggest that not only the aerobic glycolysis but also fatty acid synthesis may be involved in the subset imbalance and overproduction of IFN-γ in Th1 cells (**Figure 5D**).

In 2002, Gergely and colleagues (28) were the first to describe the metabolic changes in CD4^+^ T cells in SLE, including increased mitochondrial membrane potential (hyperpolarization) and increased ROS production. Activation of mTORC1 in CD4^+^ T cells has also been reported in a lupus mouse model as well as in SLE patients (29, 30), suggesting that differentiation to Th1 and Th17 is enhanced by mTORC1 in wild-type and autoimmune mice. Previous studies also discussed the importance of lipid metabolism in Th17 differentiation (31). Furthermore, various metabolic abnormalities have been described in CD4^+^ T cells of SLE patients, but many aspects of SLE pathology remain unknown (32, 33). Our study highlighted the pathogenic roles of IFN-γ overproduction and imbalance of T-bet^+^Foxp3^-^ and T-bet^+^Foxp3^+^ cells, and that abnormal fatty acid synthesis is an important metabolic abnormality mediating the above changes.

The functional properties of mTORC1 include enhancement of mRNA translation, ribosome biogenesis, and glycolysis in the downstream, as well as inhibition of catabolic metabolism, such as autophagy, fatty acid oxidation, and oxidative phosphorylation in the mitochondria (34, 35). Rapamycin is known as an mTORC1 inhibitor whereas 2-deoxy-D-glucose (2DG) is a glycolysis inhibitor. Studies in experimental animals have demonstrated that mTORC1 activity and inhibition of aerobic glycolysis by rapamycin and 2DG are associated with suppression of T_eff_ cell and induction of T_reg_ cells (36–38). However, the effect of rapamycin and 2DG to human CD4^+^ T cells, especially memory CD4^+^ T cells remained unclear. In addition, there is no consensus on the role of mTORC1 in the induction of T_reg_ differentiation (39, 40). In related studies, it was demonstrated that T_reg_ cells induced by selective mTORC1 deficiency by deletion of TSC and phosphatase and tensin homolog (PTEN) and persistent activation, lacked immunosuppressive capacity (40, 41). Furthermore, mTORC1 deficient mice exhibited inhibition of T_reg_ induction, setting a series of inflammatory processes (42). Several studies reported that T-bet^+^ T_reg_ cells inhibit Th1 differentiation and Th1-related autoimmunopathologies (15, 43–46), while others indicated that these cells do not possess such suppressive activity (47). Thus, fractionation of (T-bet^+^) Foxp3^+^ CD4^+^ cells includes populations of plastic and non-functional or rather pro-inflammatory cells. It is suggested that rapamycin may promote the differentiation of these cells.

It has been reported that CD45RA^-^Foxp3^lo^ non-suppressive T cells are increased in SLE patients and that these cells produce IFN-γ and IL-2 and exhibit little suppressive activity (48). On the other hand, CD45RA^-^Foxp3^hi^ cells (activated-Treg) have not been found to be consistent (48, 49). In the present study, CD45RA^-^Foxp3^lo^ non-suppressive T cells were increased in patients with SLE as previously reported (**Figure 3A**). By focusing on Th1 markers such as T-bet, IFN-γ, we found that T-bet^+^IFN-γ^+^CD45RA^-^Foxp3^lo^ non-suppressive T cells were increased, while T-bet^+^IFN-γ^-^Foxp3^hi^CD45RA^-^ activated-Treg cells were decreased (**Figures 3B, C**), and the imbalance of these subsets was caused by enhanced fatty acid synthesis (**Figures 5B, C**).

Although the mTOR signaling inhibitors, including rapamycin, have been reported to be important metabolic control drugs in SLE patients, it has limited effectiveness (50). Our study demonstrated that, unlike rapamycin, 2DG induced IFN-γ non-producing T-bet^+^Foxp3^hi^ activated-T_reg_ cells differentiation and IL-2 production. In this regard, previous studies reported that a decrease in serum IL-2 level in SLE patients is associated with inhibition of T_reg_ function with consequent enhancement of inflammatory pathology (51, 52). In fact, previous studies showed that administration of a small amount of soluble IL-2 was associated with an increase in T_reg_ cells with a resultant control of SLE disease activity (53). Evidence suggests that the primary cells that produce IL-2 are activated Th cells (54, 55). Our study demonstrated that 2DG induced the production of IL-2 from memory CD4^+^ cells in patients with SLE (**Figure 5D**), suggesting that 2DG does not only correct the abnormality of Th1 subsets through the control of abnormal cell metabolism but also improve the pathological status of SLE through the induction of IL-2 production.

Limitations of this study were that a limited number of memory CD4^+^ T cells can be isolated from the peripheral blood in patients with SLE, and therefore, detailed analysis of the mechanisms occurring in the memory CD4^+^ T cells derived from the patients was unfeasible. Furthermore, the annual incidence of SLE without treatments for the disease is too limited to allow us to perform these studies.

Taken together, SLE patients exhibited IFN-γ overproduction by Th1 cells and subset imbalance of these cells, suggesting the involvement of Th1 cells in SLE pathology such as treatment resistance. Metabolic regulators, particularly fatty acid synthesis inhibitors, may be therapeutically beneficial in SLE by correcting these abnormalities.

## Data Availability Statement

All data generated or analyzed during this study are included in this published article (and its supplementary information files).

## Ethics Statement

The studies involving human participants were reviewed and approved by the Human Ethics Review Committee of the University of Occupational and Environmental Health, Japan, and each subject provided a signed consent form (H29-045). The patients/participants provided their written informed consent to participate in this study.

## Author Contributions

SI wrote the manuscript and designed the experiments. SI, MZ, HH, GT, MH, YM, NO, YS-K, YaT, HM, MU, AN, SN, and KS performed the experiments. SI, MZ, HH, YM, and NO analyzed the data. YoT supervised the project. All authors contributed to the article and approved the submitted version.

## Funding

This work was supported in part by JSPS KAKENHI grant number #JP16K09928 and the University of Occupational and Environmental Health, Japan, through UOEH Grant for Advanced Research (#H29-903 and #H30-905).

## Conflict of Interest

KS is an employee of Mitsubishi Tanabe Pharma. SN has received speaking fees from Bristol Myers, Sanofi, Abbvie, Eisai, Eli Lilly, Chugai, Pfizer, Takeda, and also research grants from Mitsubishi Tanabe, Novartis, and MSD. YT received research grants from Mitsubishi Tanabe, Takeda, Daiichi-Sankyo, Chugai, Bristol-Myers, MSD, Astellas, Abbvie, and Eisai.

The remaining authors declare that the research was conducted in the absence of any commercial or financial relationships that could be construed as a potential conflict of interest.

## References

[1] Baechler EC Batliwalla FM Karypis G Gaffney PM Ortmann WA Espe KJ Interferon-inducible gene expression signature in peripheral blood cells of patients with severe lupus. Proc Natl Acad Sci USA (2003) 100:2610–15. 10.1073/pnas.0337679100 PMC151388 12604793

[2] Chiche L Jourde-Chiche N Whalen E Presnell S Gersuk V Dang K Modular transcriptional repertoire analyses of adults with systemic lupus erythematosus reveal distinct type I and type II interferon signatures. Arthritis Rheumatol (2014) 66:1583–95. 10.1002/art.38628 PMC4157826 24644022

[3] Tsokos GC Syst Lupus Erythematosus N Engl J Med (2011) 365:2110–21. 10.1056/NEJMra1100359 22129255

[4] Akahoshi M Nakashima H Tanaka Y Kohsaka T Nagano S Ohgami E Th1/Th2 balance of peripheral T helper cells in systemic lupus erythematosus. Arthritis Rheumatol (1999) 42:1644–8. 10.1002/1529-0131(199908)42:8<1644::AID-ANR12>3.0.CO;2-L 10446863

[5] Harigai M Kawamoto M Hara M Kubota T Kamatani N Miyasaka N Excessive Production of IFN-γ in Patients with Systemic Lupus Erythematosus and Its Contribution to Induction of B Lymphocyte Stimulator/B Cell-Activating Factor/TNF Ligand Superfamily-13B. J Immunol (2008) 181(3):2211–9. 10.4049/jimmunol.181.3.2211 18641361

[6] Caielli S Veiga DT Balasubramanian P Athale S Domic B Murat E A CD4^+^ T cell population expanded in lupus blood provides B cell help through interleukin-10 and succinate. Nat Med (2019) 25:75–81. 10.1038/s41591-018-0254-9 30478422 PMC6325012

[7] Rao DA Gurish MF Marshall JL Slowikowski K Fonseka CY Liu Y Pathologically expanded peripheral T helper cell subset drives B cells in rheumatoid arthritis. Nature (2017) 542:110–4. 10.1038/nature20810 PMC5349321 28150777

[8] Wang S Wang J Kumar V Karnell JL Naiman B Gross PS IL-21 drives expansion and plasma cell differentiation of autoreactive CD11c^hi^T-bet^+^ B cells in SLE. Nat Commun (2018) 9:1758. 10.1038/s41467-018-03750-7 29717110 PMC5931508

[9] Jenks SA Cashman KS Zumaquero E Marigorta UM Patel AV Wang X Distinct Effector B Cells Induced by Unregulated Toll-like Receptor 7 Contribute to Pathogenic Responses in Systemic Lupus Erythematosus. Immunity (2018) 49:725–39. 10.1016/j.immuni.2018.08.015 PMC6217820 30314758

[10] Rubtsova K Rubtsov AV Thurman JM Mennona JM Kappler JW Marrack P B cells expressing the transcription factor T-bet drive lupus-like autoimmunity. J Clin Invest (2017) 127:1392–404. 10.1172/JCI91250 PMC5373868 28240602

[11] Wu C Fu Q Guo Q Chen S Goswami S Sun S Lupus-associated atypical memory B cells are mTORC1-hyperactivated and functionally dysregulated. Ann Rheum Dis (2019) 78:1090–100. 10.1136/annrheumdis-2019-215039 PMC6691860 31142473

[12] Bocharnikov AV Keegan J Wacleche VS Cao Y Fonseka CY Wang G PD-1hiCXCR5- T peripheral helper cells promote B cell responses in lupus via MAF and IL-21. JCI Insight (2019) 4:130062. 10.1172/jci.insight.130062 31536480 PMC6824311

[13] Szabo SJ Kim ST Costa GL Zhang X Fathman CG Glimcher LH A novel transcription factor, T-bet, directs Th1 lineage commitment. Cell (2000) 100:655–69. 10.1016/S0092-8674(00)80702-3 10761931

[14] Lazarevic V Glimcher LH Lord GM. T-bet: a bridge between innate and adaptive immunity. Nat Rev Immunol (2013) 13:777–89. 10.1038/nri3536 PMC6290922 24113868

[15] Levine AG Medoza A Hemmers S Moltedo B Niec RE Schizas M Stability and function of regulatory T cells expressing the transcription factor T-bet. Nature (2017) 546:421–25. 10.1038/nature22360 PMC5482236 28607488

[16] Iwata S Mikami Y Sun HW Brooks SR Jankovic D Hirahara K The Transcription Factor T-bet Limits Amplification of Type I IFN Transcriptome and Circuitry in T Helper 1 Cells. Immunity (2017) 46:983–91. 10.1016/j.immuni.2017.05.005 PMC5523825 28623086

[17] O’Neill LA Kishton RJ Rathmell J. A guide to immunometabolism for immunologists. Nat Rev Immunol (2016) 16:553–65. 10.1038/nri.2016.70 PMC5001910 27396447

[18] Finlay DK Metabolic regulation of natural killer cells. Biochem Soc Trans (2015) 43:758–62. 10.1042/BST20150116 26551725

[19] O’Neill LA Pearce EJ Immunometabolism governs dendritic cell and macrophage function. J Exp Med (2016) 213:15–23. 10.1084/jem.20151570 26694970 PMC4710204

[20] Phan AT Goldrath AW Glass CK Metabolic and Epigenetic Coordination of T Cell and Macrophage Immunity. Immunity (2017) 46:714–29. 10.1016/j.immuni.2017.04.016 PMC5505665 28514673

[21] Boothby M Rickert RC Metabolic Regulation of the Immune Humoral Response. Immunity (2017) 46:743–55. 10.1016/j.immuni.2017.04.009 PMC5640164 28514675

[22] Miyara M Yoshioka Y Kitoh A Shima T Wing K Niwa A Functional delineation and differentiation dynamics of human CD4+ T cells expressing the FoxP3 transcription factor. Immunity (2009) 30:899–911. 10.1016/j.immuni.2009.03.019 19464196

[23] Park Y Jin HS Lopez J Elly C Kim G Murai M TSC1 regulates the balance between effector and regulatory T cells. J Clin Invest (2013) 123:5165–78. 10.1172/JCI69751 PMC3859395 24270422

[24] Cretney E Xin A Shi W Minnich M Masson F Miasari M The transcription factors Blimp-1 and IRF4 jointly control the differentiation and function of effector regulatory T cells. Nat Immunol (2011) 12:304–11. 10.1038/ni.2006 21378976

[25] Yang XP Jiang K Hirahara K Vahedi G Afzali B Sciume G EZH2 is crucial for both differentiation of regulatory T cells and T effector cell expansion. Sci Rep (2015) 5:10643. 10.1038/srep10643 26090605 PMC4473539

[26] DuPage M Chopra G Quiros J Rosenthal WL Morar MM Holohan D The chromatin-modifying enzyme Ezh2 is critical for the maintenance of regulatory T cell identity after activation. Immunity (2015) 42:227–38. 10.1016/j.immuni.2015.01.007 PMC4347854 25680271

[27] Roychoudhuri R Hirahara K Mousavi K Clever D Klebanoff CA Bonelli M BACH2 represses effector programs to stabilize T(reg)-mediated immune homeostasis. Nature (2013) 498:506–10. 10.1038/nature12199 PMC3710737 23728300

[28] Gergely P Jr Grossman C Niland B Puskas F Neupane H Allam F Mitochondrial hyperpolarization and ATP depletion in patients with systemic lupus erythematosus. Arthritis Rheumatol (2002) 46:175–90. 10.1002/1529-0131(200201)46:1<175::AID-ART10015>3.0.CO;2-H PMC4020417 11817589

[29] Fernandez D Perl A mTOR signaling: a central pathway to pathogenesis in systemic lupus erythematosus? Discovery Med (2010) 9:173–78. 10.1038/nrd3123 PMC3131182 20350481

[30] Yin Y Choi SC Xu Z Perry DJ Seay H Croker BP Normalization of CD4+ T cell metabolism reverses lupus. Sci Transl Med (2015) 7:274ra18. 10.1126/scitranslmed.aaa0835 PMC5292723 25673763

[31] Berod L Friedrich C Nandan A Freitag J Hagemann S Harmrolfs K De novo fatty acid synthesis controls the fate between regulatory T and T helper 17 cells. Nat Med (2014) 20:1327–33. 10.1038/nm.3704 25282359

[32] Morel L Immunometabolism in systemic lupus erythematosus. Nat Rev Rheumatol (2017) 13:280–90. 10.1038/nrrheum.2017.43 28360423

[33] Sharabi A Tsokos GC T cell metabolism: new insights in systemic lupus erythematosus pathogenesis and therapy. Nat Rev Rheumatol (2020) 16:100–12. 10.1038/s41584-019-0356-x 31949287

[34] Zeng H Chi H mTOR signaling in the differentiation and function of regulatory and effector T cells. Curr Opin Immunol (2017) 46:103–11. 10.1016/j.coi.2017.04.005 PMC5554750 28535458

[35] Pollizzi KN Powell JD Regulation of T cells by mTOR: the known knowns and the known unknowns. Trends Immunol (2015) 36:13–20. 10.1016/j.it.2014.11.005 25522665 PMC4290883

[36] Shi LZ Wang R Huang G Vogel P Neale G Green DR HIF1alpha-dependent glycolytic pathway orchestrates a metabolic checkpoint for the differentiation of TH17 and Treg cells. J Exp Med (2011) 208:1367–76. 10.1084/jem.20110278 PMC3135370 21708926

[37] Gerriets VA Kishton RJ Nichols AG Macintyre AN Inoue M Ilkayeva O Metabolic programming and PDHK1 control CD4+ T cell subsets and inflammation. J Clin Invest (2015) 125:194–207. 10.1172/JCI76012 25437876 PMC4382238

[38] Newton R Priyadharshini B Turka LA Immunometabolism of regulatory T cells. Nat Immunol (2016) 17:618–25. 10.1038/ni.3466 PMC5006394 27196520

[39] Powell JD Pollizzi KN Heikamp EB Horton MR Regulation of immune responses by mTOR. Annu Rev Immunol (2012) 30:39–68. 10.1146/annurev-immunol-020711-075024 22136167 PMC3616892

[40] Park Y Jin HS Lopez J Elly C Kim G Murai M TSC1 regulates the balance between effector and regulatory T cells. J Clin Invest (2013) 123:5165–78. 10.1172/JCI69751 PMC3859395 24270422

[41] Shrestha S Yang K Guy C Vogel P Neale G Chi H Treg cells require the phosphatase PTEN to restrain TH1 and TFH cell responses. Nat Immunol (2015) 16:178–87. 10.1038/ni.3076 PMC4297581 25559258

[42] Zeng H Yang K Cloer C Neale G Vogel P Chi H mTORC1 couples immune signals and metabolic programming to establish T(reg)-cell function. Nature (2013) 499:485–90. 10.1038/nature12297 PMC3759242 23812589

[43] Koch MA Tucker-Heard G Perdue NR Killebrew JR Urdahl KB Campbell DJ The transcription factor T-bet controls regulatory T cell homeostasis and function during type 1 inflammation. Nat Immunol (2009) 10:595–602. 10.1038/ni.1731 19412181 PMC2712126

[44] Koch MA Thomas KR Perdue NR Smigiel KS Srivastava S Campbell DJ. T-bet(+) Treg cells undergo abortive Th1 cell differentiation due to impaired expression of IL-12 receptor β2. Immunity (2012) 37:501–10. 10.1016/j.immuni.2012.05.031 PMC3501343 22960221

[45] Yu F Sharma S Edwards J Feigenbaum L Zhu J Dynamic expression of transcription factors T-bet and GATA-3 by regulatory T cells maintains immunotolerance. Nat Immunol (2015) 16:197–206. 10.1038/ni.3053 25501630 PMC4297509

[46] Tan TG Mathis D Benoist C Singular role for T-BET+CXCR3+ regulatory T cells in protection from autoimmune diabetes. Proc Natl Acad Sci USA (2016) 113:14103–08. 10.1073/pnas.1616710113 PMC5150376 27872297

[47] McPherson RC Turner DG Mair I O’Connor RA Anderton SM. T-bet Expression by Foxp3(+) T Regulatory Cells is Not Essential for Their Suppressive Function in CNS Autoimmune Disease or Colitis. Front Immunol (2015) 6:69. 10.3389/fimmu.2015.00069 25741342 PMC4332357

[48] Suen JL Chiang BL CD4^+^FoxP3^+^ regulatory T-cells in human systemic lupus erythematosus. J Formos Med Assoc (2012) Sep 111(9):465–70. 10.1016/j.jfma.2012.05.013 23021502

[49] Li W Deng C Yang H Wang G The Regulatory T Cell in Active Systemic Lupus Erythematosus Patients: A Systemic Review and Meta-Analysis. Front Immunol (2019) 10:159. 10.3389/fimmu.2019.00159 30833946 PMC6387904

[50] Perl A Activation of mTOR (mechanistic target of rapamycin) in rheumatic diseases. Nat Rev Rheumatol (2016) 12:169–82. 10.1038/nrrheum.2015.172 PMC5314913 26698023

[51] Humrich JY Morbach H Undeutsch R Enghard P Rosenberger S Weigert O Homeostatic imbalance of regulatory and effector T cells due to IL-2 deprivation amplifies murine lupus. Proc Natl Acad Sci USA (2010) 107:204–9. 10.1073/pnas.0903158107 PMC2806746 20018660

[52] von Spee-Mayer C Siegert E Abdirama D Rose A Klaus A Alexander T Low-dose interleukin-2 selectively corrects regulatory T cell defects in patients with systemic lupus erythematosus. Ann Rheum Dis (2016) 75:1407–15. 10.1136/annrheumdis-2015-207776 26324847

[53] He J Zhang X Wei Y Sun X Chen Y Deng J Low-dose interleukin-2 treatment selectively modulates CD4(+) T cell subsets in patients with systemic lupus erythematosus. Nat Med (2016) 22:991–3. 10.1038/nm.4148 27500725

[54] Malek TR The biology of interleukin-2. Annu Rev Immunol (2008) 26:453–79. 10.1146/annurev.immunol.26.021607.090357 18062768

[55] Boyman O Sprent J The role of interleukin-2 during homeostasis and activation of the immune system. Nat Rev Immunol (2012) 12:180–90. 10.1038/nri3156 22343569

