# Supplementary figures and images for: Enhanced Fatty Acid Synthesis Leads to Subset Imbalance and IFN-γ Overproduction in T Helper 1 Cells
Source: Front Immunol. 2020 Nov 30;11:593103. doi: 10.3389/fimmu.2020.593103 (PMC7734283; doi:10.3389/fimmu.2020.593103)

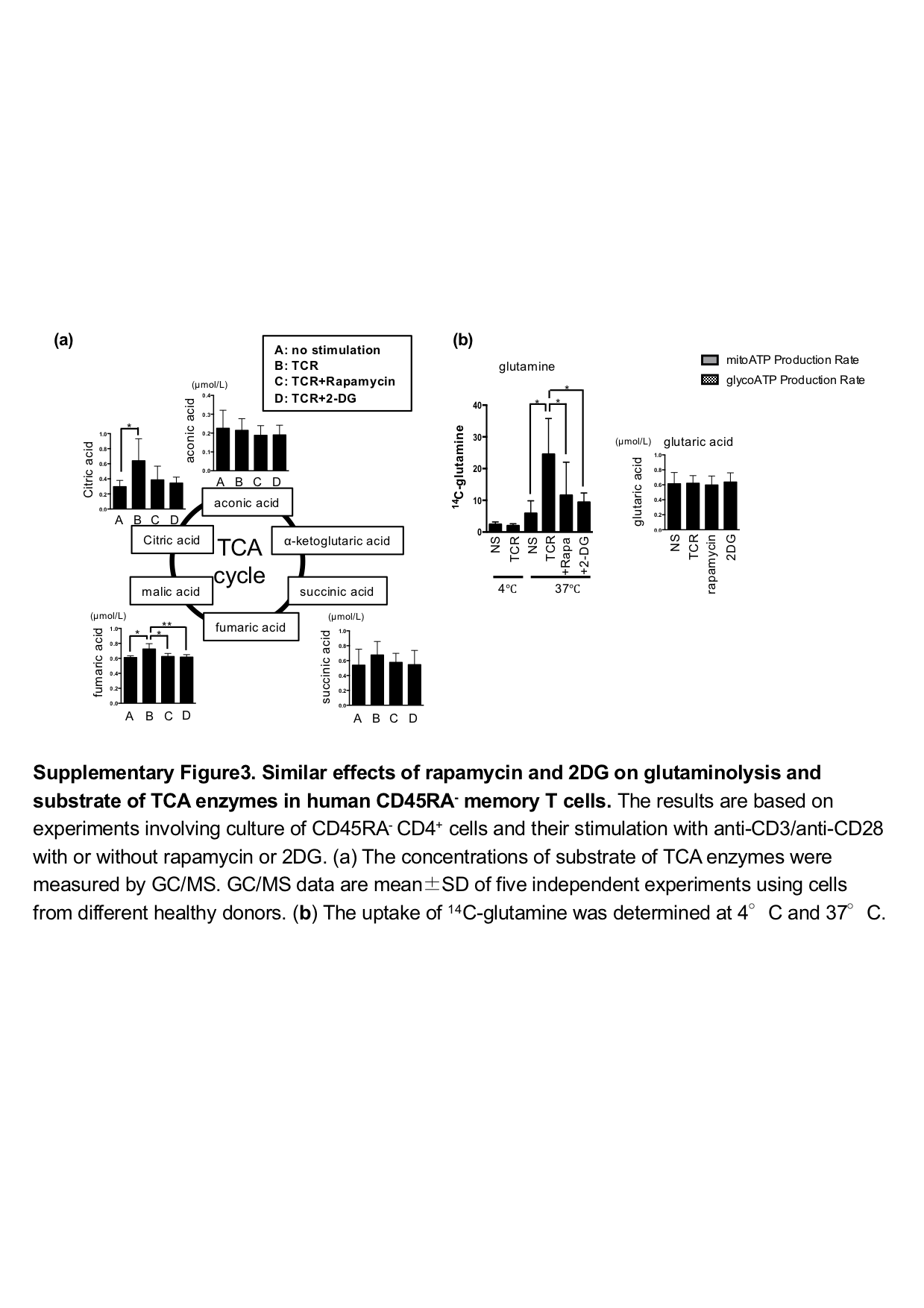

Supplement: Supplementary file 3 [file Image_3.tiff]

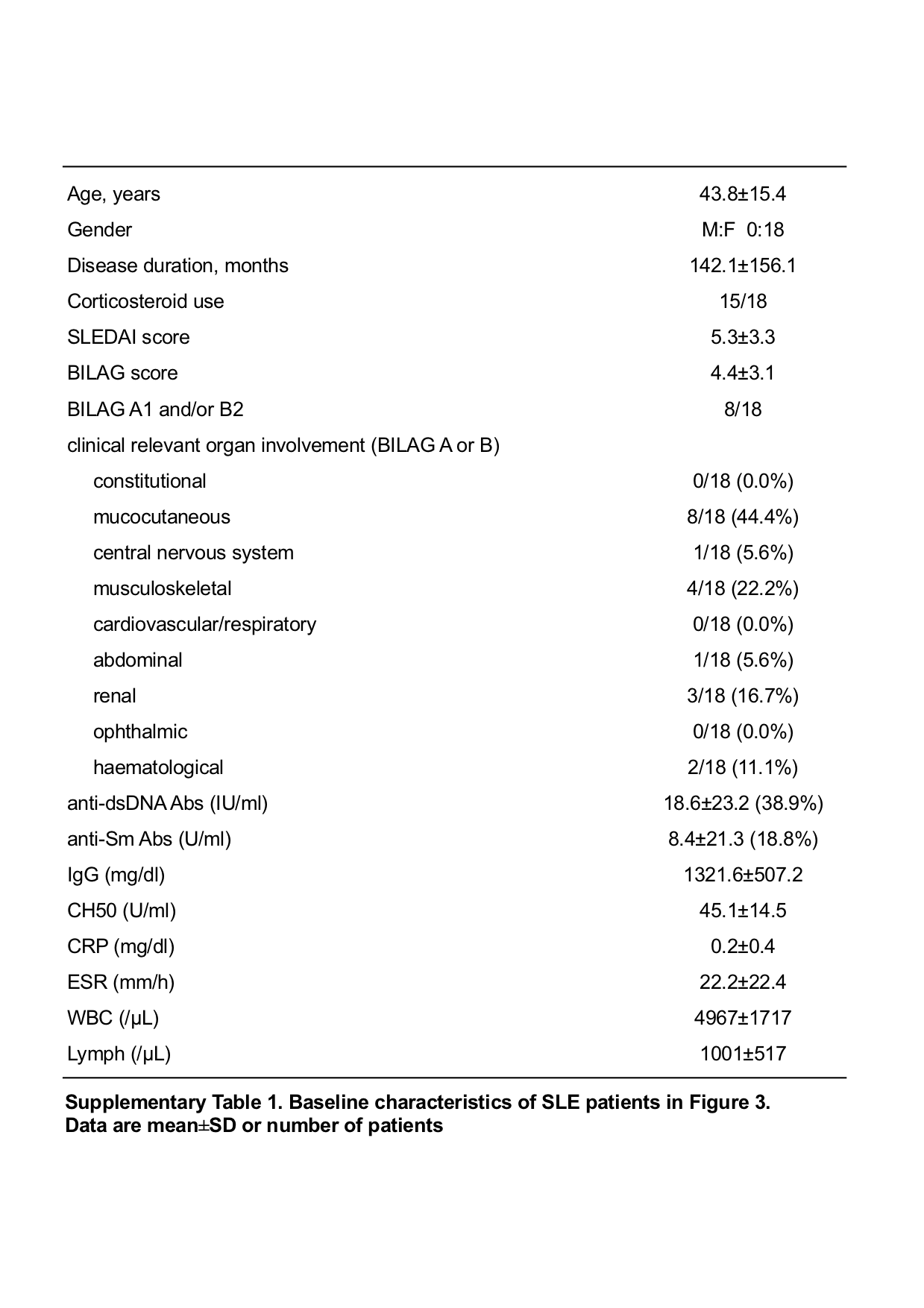

Supplement: Supplementary file 5 [file Image_5.tiff]
